# Intraspecific Aggression in Giant Honey Bees (*Apis dorsata*)

**DOI:** 10.3390/insects5030689

**Published:** 2014-09-18

**Authors:** Frank Weihmann, Dominique Waddoup, Thomas Hötzl, Gerald Kastberger

**Affiliations:** Institute of Zoology, Karl-Franzens-University Graz, 8010 Graz, Austria; E-Mails: dominique.waddoup@austrianbiologist.at (D.W.); thohoe@aon.at (T.H.); gerald.kastberger@uni-graz.at (G.K.)

**Keywords:** giant honey bee, *Apis dorsata*, defensive behaviour, shimmering waves, nestmate recognition, intraspecific and inter-colony aggression

## Abstract

We investigated intraspecific aggression in experimental nests (*expN_1_*, *expN_2_*) of the giant honey bee *Apis dorsata* in Chitwan (Nepal), focusing on interactions between surface bees and two other groups of bees approaching the nest: (1) homing “nestmate” foragers landing on the bee curtain remained unmolested by guards; and (2) supposed “non-nestmate” bees, which were identified by their erratic flight patterns in front of the nest, such as hovering or sideways scanning and splaying their legs from their body, and were promptly attacked by the surface bees after landing. These supposed non-nestmate bees only occurred immediately before and after migration swarms, which had arrived in close vicinity (and were most likely scouting for a nesting site). In total, 231 of the “nestmate” foragers (fb) and 102 approaches of such purported “non-nestmate” scouts (sc) were analysed (total observation time *expN_1_*: 5.43 min) regarding the evocation of shimmering waves (sh). During their landing the “nestmate” foragers provoked less shimmering waves (_rel_n_sh_[fb] = 23/231 = 0.0996, _rel_n_sh_[sc] = 75/102 = 0.7353; *p* <0.001, χ^2^-test) with shorter duration (D_sh_[fb] = 197 ± 17 ms, D_sh_[sc] = 488 ± 16 ms; *p* <0.001; *t*-test) than “non-nestmates”. Moreover, after having landed on the nest surface, the “non-nestmates” were attacked by the surface bees (*expN_1_*, *expN_2_*: observation time >18 min) quite similarly to the defensive response against predatory wasps. Hence, the surface members of settled colonies respond differently to individual giant honey bees approaching the nest, depending on whether erratic flight patterns are displayed or not.

## 1. Introduction

**Figure 1:**
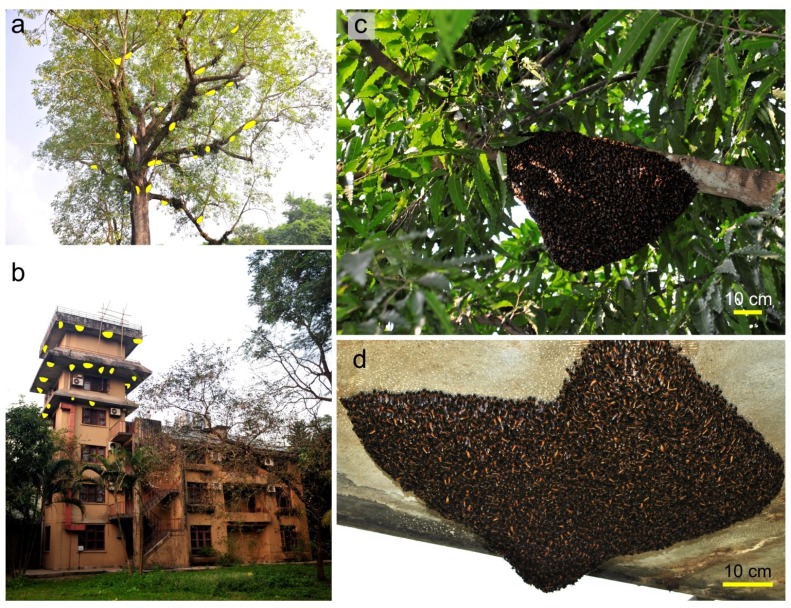
Typical giant honey bee nesting sites. Nests regularly occur on traditional sites where they form aggregations, (**a**) on a “bee tree” within the Chitwan National Park (Nepal) with 23 nests (marked in yellow); (**b**) nearby at a hotel with 18 nests; (**c**,**d**) two typically-sized, recently arrived giant honey bee swarms bivouacking on a limb of a tree (**c**), and near the final nesting site at the hotel (**d**).

The Southeast Asian giant honey bee, *A. dorsata* (Fabricius 1793), nests in the open with single-comb nests (Figure 1a,b) that hang freely from limbs, rock ledges or building structures [1]. Colonies frequently aggregate in groups of up to two hundred nests on a single tree or on a cliff site [2,3,4,5,6,7,8]. Compared to cavity-nesting honey bees such as the western honey bee (*Apis mellifera*), the giant honey bee nest is more exposed to predators and to meteorological conditions. In *A. dorsata*, the single comb of the nest is covered by a “curtain” of bees [1,9,10] that typically consists of several layers of densely clustered colony members hanging loosely attached on each other. The bee curtain has different functional regions, which change in their expression in the diurnal course: the “mouth zone” [5,9,11] is established at the sunny side of the nest, it is the interface between the outside and inside of the nest and the area where the foraging bees depart and arrive. Peripheral to the mouth zone, in the “quiescent region” [5,9,11], the curtain bees hang seemingly motionless, with their heads pointing upwards and their abdomens downwards.

Defence strategies in giant honey bee nests involving guarding. Western honey bees (*A. mellifera*) are known to defend the nest entrance to prevent invaders like wasps or foreign conspecifics [12] from entering the colony. Therefore, they feature patrolling guard bees, which pay particular attention to arriving bees with their antennae [13,14,15]. Discrimination of “non-nestmates” at the nest entrance is particularly active under robbing conditions [12,16] where foreign bees show erratic traits with characteristic hovering and sideways flight patterns [12].

Generally, guard bees form a facultative, temporal cohort of workers [12,17], which does not comprise all nestmates of the same age level [18] and not all worker bees may have been guards during their lifetimes [19]. Under threatened conditions, in particular during interspecific conflicts [20], the colony can adjust the number of guards by immediate recruitment [17]. In this context, such additional guards are termed as “defending guards” or “soldier bees” [11,21,22].

Literature about guarding behaviour in *A. dorsata* is sparse [1,7,11,23] and relates to interspecific cases. Seeley * et al.* [1] suggested that, in general, the bee curtain in *A. dorsata* consists of “inactive guards”. However, infrared investigations of *A. dorsata* nests identified two types of non-foragers [23,24] with heated-up thoraces, which may be associated with guarding behaviour: One type of “hot” bees regularly patrols the nest surface, while the second type emerges from sub-surface layers of a nest region after mechanical disturbance [11]. These latter guards are involved in a variety of collective defence traits, ranging from killing wasps by heat balling [23] to the recruitment of flying guards that are released in counter attacks against predatory birds [7].

Recognition of nestmates in Hymenoptera. Protecting the colony against non-nestmates would imply the recognition of nestmates. Such an ability is well known amongst eusocial insects [25,26] and is considered essential for social life [27].

Among western honey bees, the recognition of conspecifics is based on the detection of chemical signals such as fatty acids and alkenes [28]. These substances represent a colony-specific odour derived from a physiological genetic fingerprint [29], potentially cumulated in beeswax [30,31,32,33]. Furthermore, homing forager bees in *A. mellifera* with their heated thorax provide an enhanced release of chemical signals, which is finally presented to the guards [34] inspecting the nest entrance. Ants and other social hymenoptera use similar mechanisms [28,35,36] to classify conspecifics as nestmates or non-nestmates.

Currently, discrimination of non-nestmates in *A. dorsata* has been addressed in only two papers with contrary results. In the first paper [37], aggressive behaviour was found among conspecifics of unknown origin at artificial feeders, and in the second paper [**Error! Reference source not found.**38] worker bees originating from different nests and that gathered in an artificial compartment did not display any aggressive behaviour.

Shimmering evolved for additional collective defence. The open-nesting habit exposes *A. dorsata* to a variety of predators. Wasps are one of the major threats [11,39], which giant honey bees effectively repel from their nest by shimmering behaviour [1,2,11,39,40,41]. This defence strategy involves the immediate recruitment of hundreds of surface bees [10], which flip their abdomens upwards in a wave-like visual pattern [42]. This behaviour can be provoked by approaching individuals of different, species, such as vespine and polistine wasps, other bees (e.g., *Apis cerana*), moths and birds [1], and even nestmate foragers landing on the bee curtain. Shimmering waves are produced repetitively and display at least two categories [10,39]: (1) large-scale shimmering waves that spread over large areas of the nest surface activating hundreds of bees and are typically provoked by wasps; (2) Nestmate foragers provoke, if any, shimmering waves involving much smaller number of surface bees, which are confined to the area where the typically homing nestmate forager is expected to land.

Migration swarms. When swarms of giant honey bees abscond from established nests it is mostly due to the combined goals of migration and reproduction [43,44,45]. Weeks before absconding, the queen stops laying eggs. Just a few hours before the absconding activities, the worker bees of the swarm take honey from the comb cells and store it in their stomach. Bees scout for suitable sites [12,46] concerning the first intermediate stop (Figure 1c) [47] or the final locality (Figure 1d). Migration is considered as a response to seasonal climatic changes and to limited food resources [4,48,49,50,51]. Giant honey bees mostly migrate twice a year [4,8,52] shifting between two regions. In this context, it has been observed [43,44] that *A. dorsata* queens may return to the same traditional nest site they had occupied in previous seasons. Herein, they show remarkable abilities to time their arrival at the final nesting site in advance of the blossoming of the food plants [50]. If additional queens are present, swarming is also associated with reproduction.

Questions. We observed that *A. dorsata* colonies regularly defended their nests against conspecifics that originated from a swarm that had settled in close vicinity. Such “non-nestmates” obviously scouted on behalf of their mother swarm to find a final nesting site. We questioned under which conditions intraspecific aggression between conspecifics occurred in *A. dorsata*? Are giant honey bees capable of discriminating between “nestmates” and “non-nestmates”?

The visual pattern of shimmering [39] served as an adequate criterion to determine the ability of surface bees to discriminate between both cohorts. The shimmering strength is modified by the distance from the nest surface and by the angular velocity of the stimulating object [39], but it is also influenced by flight patterns of small moving objects in general. The discrimination hypothesis poses that surface bees at the experimental nests have the ability to discriminate between “non-nestmates” and “nestmates”. This hypothesis would be endorsed if the shimmering strength towards “non-nestmate” scouts is larger than towards homing “nestmate” foragers, analogous to the significantly more intense shimmering waves provoked by predatory wasps [11,39,41] than homing nestmates. Our findings demonstrate that giant honey bees discriminate between homing “nestmate” foragers from other conspecifics by their flight patterns. As the latter happened to originate from foreign colonies the data also prove intercolonial aggression for the first time in *A. dorsata* under the natural conditions of unmanipulated colonies.

## 2. Methods

Study sites and experimental nests (*expN_1_*, *expN_2_*). Data were collected over the course of a five-week expedition (October 25 to November 30, 2010) primarily at two typically-sized [1] *A. dorsata* colonies located in Sauraha (Chitwan, Nepal). The first experimental nest (*expN_1_*, movies 1–3) was attached to a balcony of a hotel. It was an approximately three weeks old queen-right nest with a fully developed comb of a hemispheric form (width × height: 90 × 50 cm). Interactions between the curtain bees (*expN_1_*) and scout bees, originating from a migration swarm that had bivouacked nearby (see below for identification criteria), were observed over a duration of seven days.

The second experimental nest (*expN_2_*) was a swarm cluster [12], similar to that in Figure 1d, that had newly arrived at its final nesting site. It was attached to a balcony of another hotel in Sauraha and was observed for two days (Figure 1b; movie 4). Also here, aggressive interactions among conspecifics of the settled cluster and scouts of possibly several migration swarms nearby were observed.

Video recording. In total, we collected 87 min of footage material displaying interactions between curtain bees of established *A. dorsata* nests and conspecifics identified as members of foreign swarms (see below). The high definition video camera (Panasonic HVX 200; 50 Hz) was placed in front of the experimental nests at distances between 1.5 m (*expN_1_*) and 3 m (*expN_2_*). The working distances provided an undistorted view of the nests and allowed experimental handling without disturbing the colonies. The video films were transformed into single images that totalled over 16,300 images from *expN_1_* and 15,000 images from *expN_2_*, which corresponded to experimental times of 326 s and 300 s, respectively.

“Non-nestmate” scout bees (identified as not belonging to the experimental nests). During the weeks of investigation we observed within close vicinity of the experimental nests (*expN_1_*, *expN_2_*) two incidents where old traces of wax, signifying traditional nesting sites, were inspected by a rapidly increasing number of scout bees. These scouts appeared only at those times (not earlier and not later) when these migration swarms had arrived in the surrounding area. These circumstances allowed us to identify them as “non-nestmates” in regard to the experimental colonies and thus as members of foreign migration swarms. In previous years [11,53] we had observed several similar arriving migration swarms. We also noticed bees at the experimental nests displaying erratic flight patterns that resembled those of the scouts at the wax traces. They hovered and scanned in front of the nests like wasps targeting prey, rather than bees arriving from foraging or nest members during mass flight activity [54,55,56].

**Figure 2:**
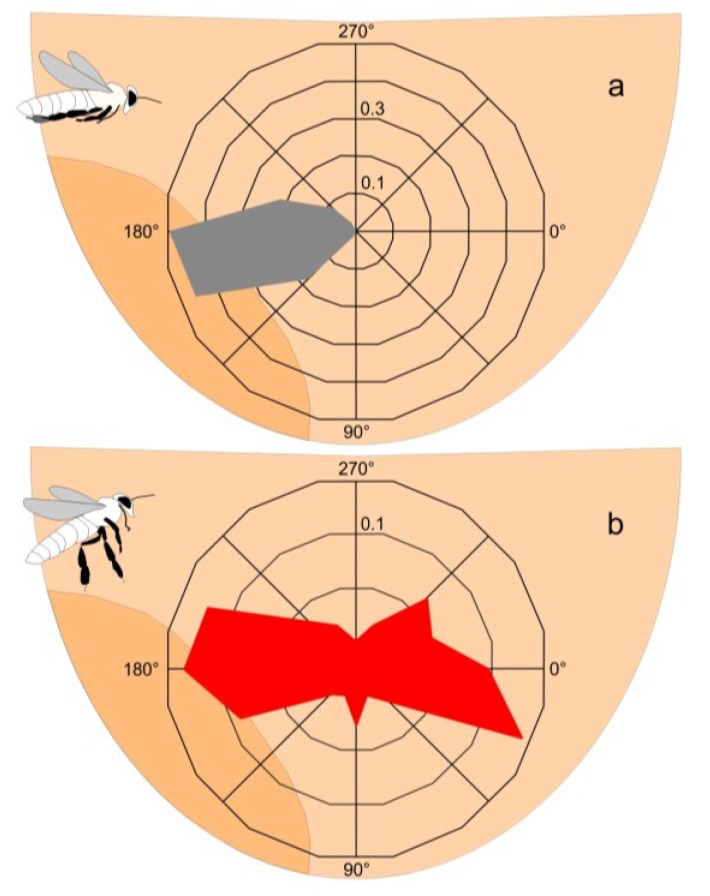
Flight characteristics of (**a**) “nestmate” forager bees (*fb*) homing to the mother nest (*expN_1_*.) and (**b**) of “non-nestmate” scouts (*sc*, originating from a migration swarm nearby) while exhibiting erratic flight patterns in front of *expN_1_*. The round diagrams illustrate the angular distributions of the flight paths as assessed in a frame-by-frame analysis; sector lines (abscissa) give the image-based flight angles (definition of directions: 0°, from the left to the right; 90°, from the top to the bottom; 180°, from the right to the left; 270°, from the bottom to the top); the ordinates show the relative number of cases. (**a**) Homing foragers flew straight towards the nest (grey plot; n_fb_ = 30), keeping the legs stretched along the body’s length axis (see scheme); (**b**) The “non-nestmate” scouts sequentially approached *expN_1_* from the left side and retreated again back to the same side (red plot; n_sc_ = 8), with their third pair of legs splayed from the body (see scheme). Light tan background area, the experimental nest (*expN_1_*): dark tan area, the mouth zone of the nest (see Movies 2,3).

Distinguishing “non-nestmate” scouts from arriving “nestmate” foragers by their flight patterns.The flight behaviour of foragers (“nestmates”) differed from that of the purported “non-nestmate” scouts. Homing foragers flew straight towards the nest, with their legs stretched along their body before landing on the bee curtain (Figure 2a; movies 1,2). The “non-nestmate” scouts had erratic flight patterns, displaying hovering and sideways flight, with sequential approach-retreatment operations and splayed their third pair of legs from the body (Figure 2b; movies 2,3). Thus, both cohorts were characterized by their flight manoeuvres, by the detail of their flight path and the orientation of the body length axis of individual bees over time (Figure 2; movies 2,3). For the latter, the positional x- and y-coordinates of the caput, thorax and the tip of the abdomen were assessed by image analysis (Image-pro, Media Cybernetics).

Detection of shimmering waves and categorizing their response status. Shimmering waves were considered if at least four adjacent bees participated in an abdominal flipping process. We measured the wave strength by the estimated number of participating bees and the wave duration (D_sh_) as the time the wave took to propagate over the nest surface. Both parameters correlate with the recruitment level of surface bees [39] in shimmering and can therefore be used to categorize the reactivity of the colony towards key stimuli such as the presence of “non-nestmate” scouts or homing foragers.

## 3. Results

Aggressive behaviours among “non-nestmate” scout bees. During our investigation period, *A. dorsata* colonies were swarming into the plain of Chitwan to stay there during the dry winter stage. However, the food supply was not yet particularly abundant; only a few plants in a nearby mustard field were in flower and provided a food source with increasing potential. In a 2000 census, the total number of giant honey bee nests found in Western Chitwan was approximately 200 [50].

At *expN_2_* we observed the arrival of 25 migration swarms within nineteen days (from October 26 to November 13, 2010; 18 of these nests are visible in Figure 1b at the hotel’s west and south front). Here, we observed severe conflicts between scout bees of seemingly different swarms, which inspected the same potential nesting sites (it is unlikely that foragers of already established colonies would be present there). Violent one-on-one fights happened between rivalling scouts that grasped one another and tried to sting each other to death. Some of them fell to the ground, where they rotated like a pegtop because of their still beating wings. On a single morning (16th November) fifteen dead pairs of such combatants were counted at this location.

**Figure 3:**
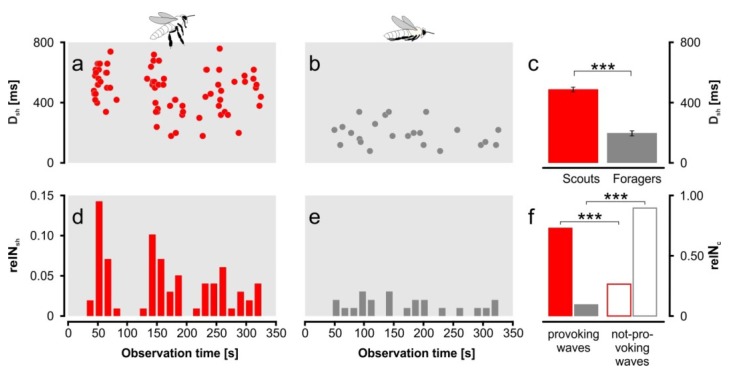
Shimmering behaviour (*expN_1_*), duration of waves (D_sh_) in ms (ordinate), provoked by (**a**) “non-nestmate” scouts (red) and by (**b**) homing “nestmate” foragers (grey) over observation time (326 s); (**c**) shimmering waves evoked by “non-nestmates” were significantly longer (means ± SEM of D_sh_; *p* < 0.001; *t*-test). Relative rates of shimmering waves (_rel_n_sh_) per 15 s intervals provoked by (**d**) “non-nestmates” and by (**e**) foragers over observation time; (**f**) scouts more often provoked shimmering waves (solid bars), but the relative number of approaches (outlined bars) without provoking shimmering waves was much lower. Stars indicate the significance levels (*p* <0.001; χ^2^-test) between the relative numbers (sc, fb).

Aggressive behaviours of guards of settled colonies against “non-nestmate” scout bees. We investigated interactions between two established colonies and “non-nestmates” that had been identified as scout bees of foreign swarms (for identification of “non-nestmates”, see Methods). The first colony (*expN_1_*, movie 1) had brood and stored food, while the second colony (*expN_2_*, movie 4) had recently arrived and, therefore, featured a cluster-like nest structure (Figure 1d) with only a small central comb. At both experimental nests (*expN_1_*, *expN_2_*) three forms of interactions (a–c) were observed.

**Figure 4:**
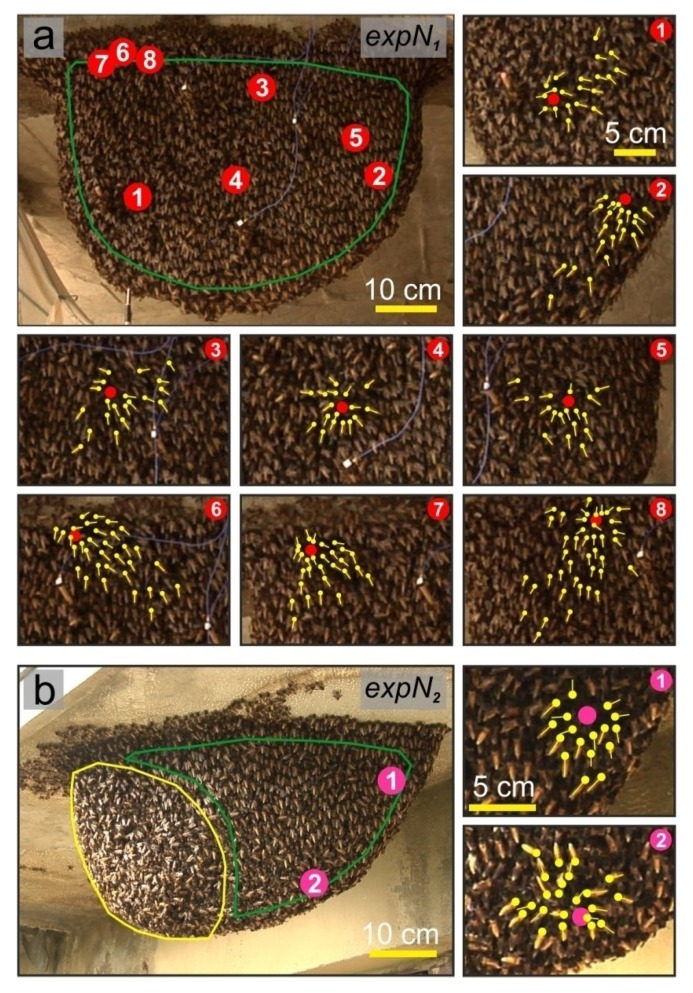
Mobilisation of guards against “non-nestmate” scout bees in *expN_1_* (**a**) and *expN_2_* (**b**). In the mouth zone (sketched by the open yellow polygon, only developed in *expN_2_*) foraging bees depart and arrive, while the quiescent zone (green polygons) is the largest part of the bee curtain where the bees are positioned with their heads up and the abdomens down; the complementary areas are the rim zones at the sides and the attachment zone on the top (not marked). Red full circles in panel a (see subpanels a1–a8) and (**b**) magenta full circles in panel b (see subpanels b1–b2) refer to the episodes and places where scouts had landed. Immediately after landing, guards grabbed the invader and others collectively moved towards the invader. Yellow dots mark the thoracic positions of the guards, yellow lines mark the direction of their long body axis (see movies 1,4,5).

(a) “Non-nestmate” scout bees scanned around the settled nests and landed on the concrete above the attachment areas of the nests (*n* = 11). They were attacked by guards that had crawled up from the established nests below.
(b) When such “non-nestmates” scanned in front of the experimental nests they provoked massive, repetitive shimmering waves (D_sh_ = 488 ± 16 ms; Figure 3a,d). In contrast, arriving “nestmate” forager bees rarely evoked shimmering waves (movie 1, Figure 3b,d) and, if they did, the waves were significantly smaller and shorter (D_sh_ = 197 ± 17 ms; *p* <0.001; *t*-test). In detail, in the observation window of 326 s we recorded 231 landings by forager bees [fb] and 102 approaches by “non-nestmate” scout bees [sc]. Forager bees evoked shimmering waves [sh] in only 23 cases (_rel_n_sh_[fb] = 23/231 = 0.0996), while “non-nestmate” scouts caused a significantly higher relative number of shimmering waves (_rel_n_sh_[sc] = 75/102 = 0.7353; *p* < 0.001; χ^2^-test; Figure 3f).
(c) While flying in front of the investigated nests, “non-nestmate” scouts occasionally touched the nest surface or tentatively landed there (24 observations at *expN_1_* and *expN_2_*; over 18 min). In these cases, the experimental colony mobilised a cohort of surface bees nearest to the scout bee in a fraction of a second (movies 4,5). These guards immediately twisted around the invader and tried to seize her, while additional guards moved towards this site of the nest surface to join the collective defence action (24.63 ± 3.05 guards; *n* = 8 scout landings; Figure 4a,b). Lastly the invader was hauled within deeper layers of the bee curtain and stung to death. The dead bodies were subsequently removed from the bee curtain. In contrast, homing “nestmate” forager bees landed on the bee curtain, started dancing, crawled into the bee curtain and remained unbothered by the guards of the experimental nests (movies 1,4,5).

## 4. Discussion

The field observations addressed in this paper provide evidence for intraspecific and intercolonial aggression in giant honey bees (*Apis dorsata*). In *A. dorsata*, both aspects, aggression among conspecific non-nestmates and nestmate recognition, have until now not been investigated under natural conditions. Breed * et al.* [38] tested nestmate recognition in *A. dorsata* by using bioassays under experimental compartment conditions. They suggested, with particular reference to the western honey bee *A. mellifera*, that large food stores together with the propensity of colonies to rob food resources from other colonies [57], have promoted the evolution of nestmate recognition [58]. As the authors could not observe aggression among *A. dorsata* non-nestmates, nor could they prove nestmate recognition, they assumed that *A. mellifera* is unique among honey bees regarding intraspecific aggression [38].

However, conforming to the arguments of Breed * et al.* [38], the evolution of nestmate recognition should also have evolved in giant honey bees (*A. dorsata*, *A. laboriosa*). In particular, the following behavioural traits should have promoted nestmate recognition: giant honey bee colonies (a) comprise large honey stores of up to 45 kg [5], which is a considerable amount in comparison to the honey stores of wild nesting *A. mellifera* colonies [59]; (b) they may occur in a high density of colonies at aggregation sites [2,6,8,43,44] and (c) their open nesting habit entails a high likelihood of invasion by insect nest parasites, such as other bee species and wasps, and, at least theoretically, also makes them susceptible to robbing by conspecifics.

Potential nest parasites like Eastern honey bees (*Apis cerana*) or wasps, such as the yellow paper wasp (*Polistes olivaceus*), regularly occur at *A. dorsata* nests. When the defence status of an *A. dorsata* colony is weakened and the bee curtain is thinned out, the chances of potential parasites feeding on the open honey cells or on the larvae of uncapped cells increases. Non-nestmate conspecifics may try to enter the foreign nest during drifting [29,60] or in a collective action of robbing [22]. Robbing behaviour is unusual in giant honey bees; we have never observed such behaviours at *A. dorsata* nests, neither during arrival of migration swarms at traditional nesting sites, nor during the prospering period of aggregated nests. However, the mission of non-nestmate scouts of a recently arrived mother swarm to search for a nesting site, for which we presented the data in the paper, may also comprise preparations for a forthcoming amalgamation [49] of such a swarm with another queen-right colony. Throughout years of observations, we noticed in a series of cases that some migration swarms regularly merge together after having established at a traditional nesting site within close vicinity to each other. Within days, the final amalgamated swarm had increased to double its size while the original number of swarms had decreased at the same site.

Aggressive behaviours among scout bees from seemingly different swarms. Hundreds of *A. dorsata* swarms regularly immigrate to a confined region, such as the plains of Chitwan, in a limited seasonal period. The large rate of arriving colonies may lead to strong competition for high-quality nesting sites (cf. [61]), which often correspond to traditional locations where hundreds of nests may aggregate [2,6,8,43,44]. Sometimes, several *A. dorsata* swarms may simultaneously explore a single potential nesting site, which likely happens during the final phase of migration. This may give rise to aggression among their scouts at such places, similar to *A. mellifera*, as swarms defend the nesting site selected for house-hunting against competing swarms [61].

In *A. dorsata*, such aggressive interactions may culminate in extremely challenging fights, where the participants kill each other. That way, they would not be able to re-join their mother swarms, but they would have successfully prevented, to some extent, the competing colony from settling down at the expected site. In this way, the aggressive scouting behaviour can be taken as a further example of extreme altruism [62] in insect societies.

Aggressive behaviours of a settled colony against conspecifics displaying erratic flight patterns. In this paper, scout bees hovering and scanning around an established nest were identified as “non-nestmates”. The characteristic features (see Methods) were time of occurrence and erratic flight patterns, compared to homing foragers and, additionally, similar to the behaviour of *A. mellifera* guards against potential robber bees [12], the quick response of surface bees with ferocious attacks (movies 1,4,5) after their landing on the bee curtain.

It is well known that surface bees on *A. dorsata* nests are capable of distinguishing nest parasites, such as flying specimens of *A. cerana* that approach the nest and try to enter the bee curtain to feed on uncapped honey cells [1] and also predatory birds (*i.e.*, honey buzzards) approaching the nest from a distance of 20 m (personal observations). This kind of responsiveness to external cues becomes evident particularly because the colonies adjust the repetition rate and recruitment in shimmering waves [39] regarding the specific threat. Butterflies, beetles, or even sparrows and pigeons, which are obviously harmless to giant honey bees, but nevertheless pass closely to their nests, trigger only a few, small and short shimmering waves, whereas harmful wasps or harmful birds elicit more repetitive and stronger waves [1,39].

In this context, it is not astonishing that surface bees of *A. dorsata* nests also differentiate between homing foragers and “non-nestmate” scouts solely by their flight patterns (Figure 2; movies 2,3; [12]). Remarkably, the graded shimmering response occurs well before the respective landing operations are being processed. We estimate that the latency period between the moment of the visual detection of the flying object (by the straight flight of a homing nestmate, the erratic flight pattern of a flying wasp, or that of a “non-nestmate” scout) and the onset of the shimmering reaction is not more than 80 ms [41]. Thus, shimmering is here taken as a statistical colony response signalling, in particular for the nestmates in the bee curtain [42], harmlessness or harmfulness of the objects discerned.

Lastly, these findings provide evidence of the capacity of giant honey bees to discriminate between nestmates and non-nestmates (Figure 3 and Figure 4; movies 1,4,5), at least in the defence contexts concerning predatory wasps and the scouts of foreign colonies which inspect potential nesting sites. The bees of the nest surface responded to homing nestmate foragers with less frequent and weaker shimmering than to (Figure 3) “non-nestmate” scouts, which resembles the massive response of *A. dorsata* colonies towards wasps [39]. If such intruders eventually land on the bee curtain of a target nest, their status as “non-nestmates” might be decided by the detection of odours [12], such as from fatty acids and alkenes, as found for the western honey bee [28]. Additionally, the higher temperature of the thorax of flying bees may enhance the release of these substances facilitating the recognition by the inspecting guards [34].

Polyphenic capacity for defensiveness. Giant honey bee colonies can respond quickly to short-term perils. The reason for this potential for rapid adequate response is that the cohorts of curtain bees in a giant honey bee nest peripheral to the mouth zone are engaged in multiple tasks [63,64,65,66]. Each individual is, for instance, able to participate in abdominal flipping, keep up additional honey stores in their honey stomachs [5,11], perform fanning behaviour in convection funnels or, particularly when in a surface position, recognize external perils by the visual display of their adversaries. Environmental needs determine here the momentary deployment of any of these polyphenic abilities of a worker bee. For instance, when a “non-nestmate” scout has landed on the nest surface, the ratio of guards at the landing position may increase by a recruitment of “soldier” bees from different positions of the nest surface or even from subsurface layers [17,20,22,66]. Hereby, a number of bees at the nest surface switch from a seemingly quiescent state to a defensive guarding role. Another polyphenic trait of *A. dorsata* concerning defence recruitment was observed [67] when cohorts of surface bees transformed from flickering mode (represented by collective, stochastic abdomen flipping) to shimmering mode [11,41,67].

The equilibrium between colony defence and colony aggregation. When settled colonies chase scouts from competing colonies away from their nesting site they benefit with their selfish behaviour by protecting their own colony. This behaviour particularly favours established colonies because it retains their position by restricting competition for nesting sites and food supply. However, giant honey bees are famous for large, potentially dense aggregations, which may encompass more than a hundred colonies at a single, typical traditional nesting site (Figure 1a,b) [60,68]. Interestingly, migration swarms decide to nest at certain places due to the principle of *first come first serve*, but nevertheless allow nesting of other swarms within their close vicinity. Formerly competing colonies may accept each other within days and the nests, particularly those on tree limbs, may be built only a few centimetres apart [44,53,60]. The reason for this change of acceptance is that when the first swarms arrive at the nesting sites the food is still scarce, but within a few days to weeks the mustard fields blossom and food is more than abundant.

Lastly, the occurrence of a colony aggregation provides superordinate strategies of collective defence, particularly against predatory birds [7,53] such as the bee eater (*Nyctyonis athertoni*) or the honey buzzard (*Pernis* spec.), while also allowing colonies to host drifted worker bees from neighbouring nests [60]. Such changes in the acceptance of unrelated conspecifics are also found in *A. mellifera* [29] and in other eusocial Hymenoptera [69].

In summary, our findings provide evidence that individual bees and the bee curtain of *A. dorsata* are capable of distinguishing “nestmates” from “non-nestmates”. This was indicated by the contrasting shimmering responses, which differ between homing nestmate foragers and obvious scouts of migration swarms that arrive in the vicinity of the experimental nests, inspect potential nesting sites and display erratic flight patterns. The responses of surface bees against “non-nestmate” scouts resemble the defence traits displayed against preying wasps and range from stronger shimmering waves [1,2,11,39] to heat balling [23].

## Supplementary Materials

**Movie 1.** Responses of guards of a settled colony against “non-nestmate” scouts, “nest-mate” foragers and a preying wasp. This movie shows the response by members of the experimental nest *expN_1_* to a “non-nestmate” scout bee landing (thorax marked with a red dot) on the bee curtain. In the first seconds of the film, the scout bee scans the front of the nest and initiates shimmering waves before landing on the bee curtain. Immediately after landing, guards grab the invader and ball around it, while shimmering activity continues. In this phase, the red circle marks the approximate position of the guard hidden in the bee curtain. The movie also shows additional “non-nestmate” scout bees (thoraces marked with yellow dots) scanning the front of the nest without making physical contact, but which provoke shimmering waves. In contrast, homing “nestmate” forager bees (marked green) rarely provoke shimmering waves and were never attacked by guards. In addition, a wasp specimen (*Vespa affinis*; marked light blue) also passed by and provoked shimmering waves along the lower nest rim.

**Movie 2.** Flight characteristics of a homing “nestmate” forager bee before and during the landing operation. Homing “nestmate” forager bee (thoraces marked by green dots) flying straight towards the nest rarely provokes shimmering waves and is unnoticed by guards. Small green dots denote the thorax positions at the frame time; the short yellow lines show the orientation of the abdomen and the continuous green line gives the flight path of the ten previous frames.

**Movie 3.** Typical flight characteristics of a non-nestmate scout bee. A “non-nestmate” scout bee (thorax marked by a red dot) originating from a migration swarm, which had bivouacked nearby, displaying a scanning flight in front of *expN_1_* and initiating shimmering waves. Her flight pattern is characterised by approaching and moving away from the nest (cf. Figure 2). For further details, see caption of movie 2.

**Movie 4.** How surface bees of the established nest seize the “non-nestmate” scout. Two “non-nestmate” scout bees, whose positions are roughly marked by red circles, hover closely in front of the nest. The surface bees nearest to the scout, even in the quiescent region of the bee curtain, twist around and seize the scout, hauling it into the bee curtain where such invaders are usually stung to death. In both cases, the film shows how the “non-nestmate” scout bees escape.

**Movie 5.** How guards move towards “non-nestmate” scout bees that land on the nest surface. The movie displays the unique collective movement of guards, initiated by the landing of a “non-nestmate” scout on *expN_1_*. Afterwards, some guard bees grab the invader, while others move towards the landed scout bee to join the attack (for further information, see Figure 4).
